# Effect of body mass index on survival in patients with metastatic colorectal cancer receiving chemotherapy plus bevacizumab: a systematic review and meta-analysis

**DOI:** 10.3389/fnut.2024.1399569

**Published:** 2024-07-16

**Authors:** Xiaohui Lang, Chengliang Tong, Yang Yu, Huiyan Li

**Affiliations:** ^1^Department of Gastrointestinal Oncology, Harbin Medical University Cancer Hospital, Harbin, Heilongjiang, China; ^2^Department of Nursing, Harbin Medical University Cancer Hospital, Harbin, Heilongjiang, China

**Keywords:** body mass index, survival, metastatic colorectal cancer, bevacizumab, meta-analysis

## Abstract

**Aim:**

This systematic review and meta-analysis was to evaluate the relationship between body mass index (BMI) and the clinical outcomes in patients with metastatic colorectal cancer (mCRC) undergoing treatment with bevacizumab plus chemotherapy.

**Methods:**

The search for relevant literature was conducted across PubMed, Embase, Cochrane Library, and Web of Science, with the final search date being October 4, 2023. We utilized the weighted mean differences (WMDs), risk ratios (RRs), or Hazard ratios (HRs) as the metric for effect sizes, which were accompanied by 95% confidence intervals (CIs).

**Results:**

A total of 9 studies were included for analysis. The results indicated that non-obese patients with mCRC undergoing treatment with bevacizumab experienced a reduced overall survival (OS) at the six-month compared to their obese counterparts (RR: 0.97, 95% CI: 0.94 to 1.00, *p* = 0.047). Furthermore, no significant differences in one-year, two-year, and five-year OS, as well as PFS and median OS, were observed between obese and non-obese mCRC patients undergoing treatment with bevacizumab plus chemotherapy.

**Conclusion:**

These findings suggest that obesity may play a role in the short-term OS of patients with mCRC undergoing bevacizumab treatment. The clinical implications of these findings underscore the importance of considering patients’ BMI in the context of mCRC care. This study may also help guide personalized treatment strategies and further research into the interplay between obesity, treatment efficacy, and patient survival in mCRC. However, further investigation is warranted to substantiate the findings of this study.

## Introduction

Colorectal cancer (CRC) stands as one of the most prevalent malignancies worldwide (1) and constitutes the second leading cause of cancer-related mortality in the United States (2). Metastasis is a common occurrence in colorectal cancer, affecting approximately 50% of CRC patients (3). Metastatic colorectal cancer (mCRC) remains a lethal condition, with a 5-year survival rate of approximately 14% (4). Patients with mCRC typically undergo surgery, or a combination of surgery and radiotherapy and/or chemotherapy, as their primary treatment approach (5). Concurrently, targeted therapies such as bevacizumab have been developed (6). Bevacizumab is typically used in conjunction with chemotherapy agents, significantly improving the overall survival (OS) of mCRC patients (5). Despite the utilization of various therapeutic approaches, patients diagnosed with mCRC continue to exhibit a poor prognosis (2). Therefore, it is essential to investigate the factors that influence the survival of mCRC patients receiving bevacizumab.

The impact of obesity as a risk factor on the development of CRC is well-established (7–9). Several studies reported that patients with higher body mass index (BMI) had an increased risk of CRC mortality (10, 11). However, previous studies also indicate that being overweight is associated with reduced mortality or prolonged survival in patients with CRC or mCRC (12, 13), a phenomenon referred to as the “obesity paradox” (12). Bevacizumab is an anti-tumor drug that belongs to a class of drugs called anti-vascular endothelial growth factor (VEGF) drugs (14). Moreover, obesity is associated with increased VEGF levels (15). Therefore, the effectiveness of bevacizumab treatment may vary depending on the patient’s BMI (16). There is speculation that the prognosis of mCRC patients receiving bevacizumab may also be related to BMI. In second-line treatment, patients with mCRC undergoing chemotherapy with bevacizumab who have a higher BMI exhibit improved prognosis regarding progression-free survival (PFS) and OS compared to those with normal weight (17). However, in the study by Artaç et al. (16), among patients undergoing treatment with bevacizumab-based regimens, the prognosis may be poorer for obese individuals compared to non-obese patients with mCRC. A pooled analysis revealed that BMI was not a prognostic factor for PFS and OS in mCRC patients treated with triplet therapy in addition to bevacizumab (18). To date, there remains controversy regarding the association between BMI and the prognosis of mCRR patients receiving bevacizumab, thereby, a meta-analysis is highly warranted.

Herein, this systematic review and meta-analysis aims to assess the association between BMI and the prognosis of mCRC patients receiving bevacizumab. A meta-analysis can provide a more robust estimation of effect sizes and help address any heterogeneity or inconsistencies among the available studies, thus offering valuable insights into the clinical implications of BMI in mCRC patients treated with bevacizumab.

## Methods

This meta-analysis adhered to the PRISMA (Preferred Reporting Items for Systematic Reviews and Meta-Analyses) guidelines (19).

### Search methods for identification of studies

The databases searched included PubMed, Embase, Cochrane Library, and Web of Science, with the search period ending on June 07, 2024. The search strategy was followed using the PICOS framework. The Pubmed search strategy was as follows: “Body Mass Index” OR “Index, Body Mass” OR “Quetelet Index” OR “Index, Quetelet” OR “Quetelet’s Index” OR “Quetelets Index” OR “BMI” AND “Colorectal Neoplasms” OR “Colorectal Neoplasm” OR “Neoplasm, Colorectal” OR “Neoplasms, Colorectal” OR “Colorectal Tumors” OR “Colorectal Tumor” OR “Tumor, Colorectal” OR “Tumors, Colorectal” OR “Colorectal Cancer” OR “Cancer, Colorectal” OR “Cancers, Colorectal” OR “Colorectal Cancers” OR “Colorectal Carcinoma” OR “Carcinoma, Colorectal” OR “Carcinomas, Colorectal” OR “Colorectal Carcinomas.” The complete search strategies for other databases are detailed in Supplementary File 1. The retrieved literature was imported into EndNote X9 for initial screening based on reading titles and abstracts. After the initial screening, articles were further assessed by reading the full text to exclude those that did not meet the criteria. Finally, the remaining articles were included in this study.

### Inclusion and exclusion criteria

The inclusion criteria were structured in accordance with the Population, Intervention, Comparator, Outcome, and Study design (PICOS): (1) population: patients with mCRC undergoing treatment with bevacizumab; (2) intervention and comparator: BMI classifications (Underweight for a BMI less than 18.5 kg/m^2^, normal weight for a BMI between 18.5 and 24.9 kg/m^2^, overweight for a BMI from 25 to 29.9 kg/m^2^, and obesity for a BMI of 30 kg/m^2^ or higher) in mCRC patients undergoing treatment with bevacizumab; (3) outcome: OS and PFS; (4) study design: cohort studies; (5) studies published in English language.

Exclusion criteria: (1) animal experiments; (2) case reports, conference abstracts, letters, protocols, editorial materials, guidelines, and expert consensus; (3) studies unrelated to the topic.

### Data collection

Two investigators (Xiaohui Lang, and Chengliang Tong) independently extracted the following data from eligible studies: the name of the first author, year of publication, country, study design, population, BMI classifications, age (year), gender, site of primary tumor, treatments, metastases, Kras status, follow up (months), and outcomes. If disagreements arose, they were resolved by a third investigator (Yang Yu).

### Assessment of quality of studies

For the quality assessment of the included studies, the modified Newcastle-Ottawa Scale (NOS) (20) scoring system was utilized. The scale has a total score of 9 points, with scores ranging from 0 to 3 considered low quality, 4 to 6 medium quality, and 7 to 9 high quality. The NOS scale consists of three major items: “Selection,” “Comparability,” and “Outcome.” Each study could receive a maximum of one star for each item in “Selection” and “Outcome,” and a maximum of two stars for “Comparability.”

### Statistical analysis

All studies conducted statistical analyses using Stata 15.1 software. For measurement data, weighted mean differences (WMDs) were used as the effect measure, while for enumeration data, risk ratios (RRs) were employed. Studies providing hazard ratios (HRs) were directly combined, and effect sizes were presented with 95% confidence intervals (CIs). Heterogeneity testing was performed for each outcome, with I^2^ values ≥50% indicating significant heterogeneity, and analyzed using a random-effect model; otherwise, a fixed-effects model was utilized. We have reassessed the appropriateness of the fixed-effects model over the random-effects model due to the limited number of studies included in a meta-analysis. Sensitivity analyses were conducted for all outcomes. A publication bias assessment was conducted for the results of over 10 papers. A significance level of *p* < 0.05 was considered statistically significant.

## Results

### Study selection process and characteristics of included studies

**Figure 1:**
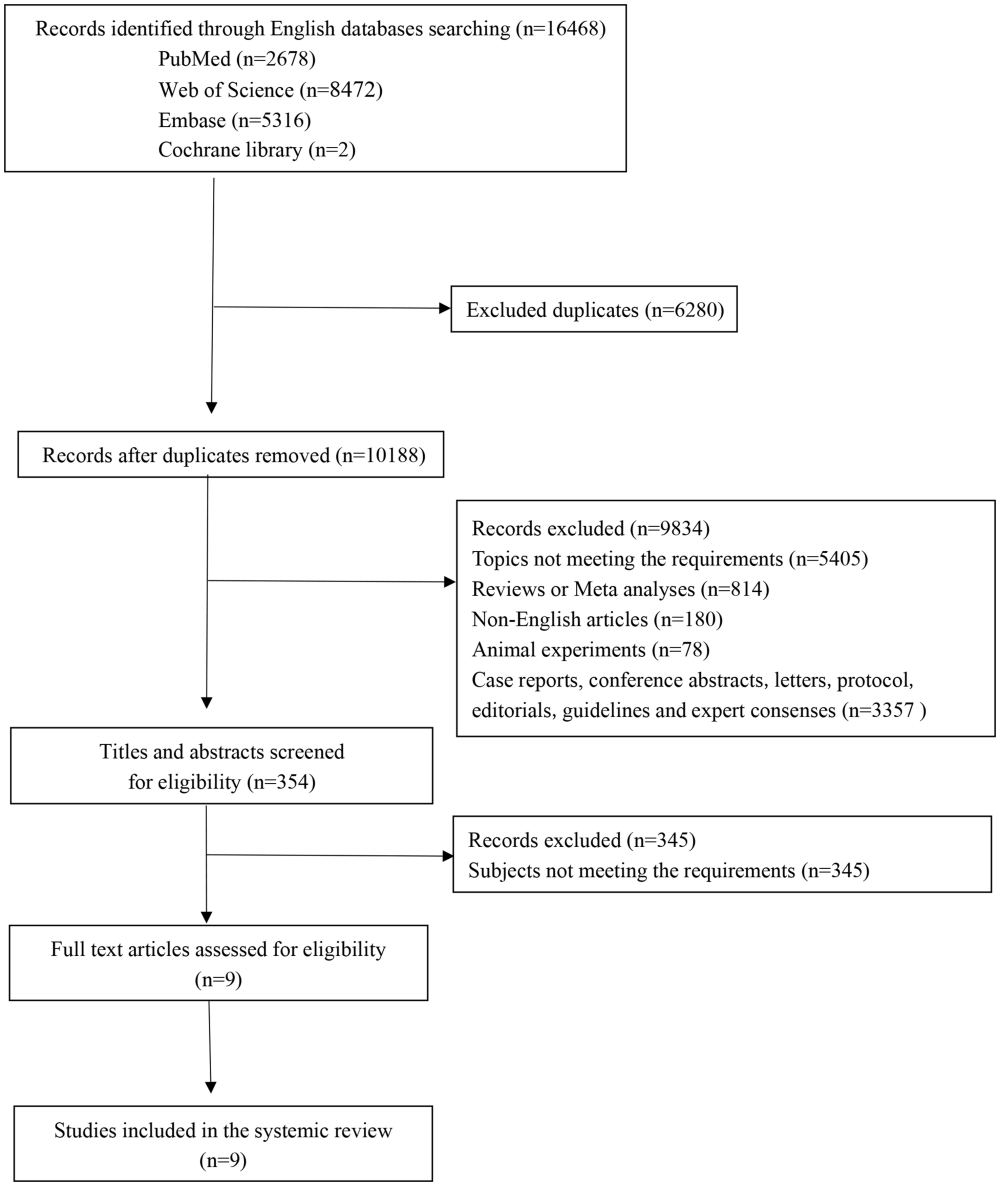
The flow of the study selection process.

The initial search across multiple databases yielded 16,468 records, comprising 2,678 from PubMed, 5,316 from Embase, 8,472 from Web of Science, and 2 from Cochrane. After deduplication, the number of records was narrowed down to 10,188. A focused screening of titles and abstracts whittled the pool to 354 potentially relevant records. Subsequently, a thorough review of full-text articles was conducted, resulting in 9 studies deemed suitable for inclusion. Ultimately, these 9 studies (16–18, 21–26) were incorporated into the meta-analysis. The flow of the study selection process is depicted in Figure 1. Across the 9 studies included, a cumulative total of 2,814 patients were included for analysis. The included studies were published over a span of 13 years, from 2009 to 2022. The quality assessment results indicated that nine studies were categorized as high quality. Table 1 presents the characteristics of the included studies.

**Table 1:** Basic characteristics of included studies.

Author	Year	Country	Study design	Population	Groups	Sample size (*N*)	Age (year)	Male/Female	Site of primary tumor	Treatments (*N*)	Metastases (*N*)	Kras status (*N*)	Follow up (months)	Outcomes	QA
Artac	2019	Turkey	Cohort study	MCRC patients who received bevacizumab-based chemotherapies	BMI <30	474	58 (33.68–82.32)	299/175	NA	FI + BEV: 308; FO + BEV: 142; F + BEV: 24	Liver: 215; Multiple: 147; The other sites: 110	Wild: 163; Mutant: 157	>50	PFS, OS	8
					BMI >30	89	58 (38.04–77.96)	36/53	NA	FI + BEV: 52; FO + BEV: 33; F + BEV: 4	Liver: 52; Multiple: 33; The other sites: 4	Wild: 37; Mutant: 26	>50		
Aykan	2013	Turkey	Cohort study	Patients with metastatic colorectal adenocarcinoma treated with fluoropyrimidine-based combination chemotherapy plus bevacizumab	BMI <25	21	60.5 (34–78)*	32/22	NA	Oxaliplatin+BEV: 19; Irinotecan+BEV: 35	NA	Mutant: 29; Wild: 25	10 (3–57)*	PFS	7
					BMI ≥25	33			NA		NA		10 (3–57)*		
Cong	2018	China	Cohort study	Patients with stage IV CRC treated with fluoropyrimidine-based combination chemotherapy plus bevacizumab	Low △BMI group	114	62.25 ± 11.39	68/46	NA	NA	Lymph nodes metastasis: 48	NA	25 (3–78)*	PFS, OS	8
					High △BMI group	110	62.25 (40.03–84.47)	64/56	NA	NA	Lymph nodes metastasis: 49	NA	25 (3–78)*		
Cybulska-Stopa	2020	Poland	Cohort study	All patients had histopathologically confirmed colon cancer and received bevacizumab with FOLFOX chemotherapy (oxaliplatin, 5-Fluorouracil, leucovorin) as second-line treatment	BMI <25	74	63 (34–80)*	27/47	NA	NA	NA	Wild: 22; Mutated: 17; Not available: 35	>25	OS	7
					25 ≤ BMI <30	109	66 (41–82)*	69/40	NA	NA	NA	Wild: 29; Mutated: 29; Not available: 51	>25		
					BMI ≥30	54	65 (43–78)*	32/22	NA	NA	NA	Wild: 23; Mutated: 15; Not available: 16	>25		
Dell-Aquila	2022	Italy	Cohort study	mCRC patients receive FOLFIRI plus bev, FOLFOXIRI plus bev, FOLFOX plus bev, FOLFOXIRI plus bev	BMI <18.5	52	57 (29–73)*	14/38	Right: 17; Left and rectum: 35; NA: 0	Chemotherapy+BEV	NA	RAS mut: 33	>40	PFS, OS	8
					18.5 ≤ BMI <29.9	952	60 (29–75)*	565/388	Right: 340; Left and rectum: 588; NA: 24	Chemotherapy+BEV	NA	RAS mut: 541	>40		
					BMI >30	156	62 (33–75)*	95/61	Right: 43; Left and rectum: 111; NA: 2	Chemotherapy+BEV	NA	RAS mut: 92	>40		
Guiu	2009	France	Cohort study	Metastatic colorectal adenocarcinoma patients were given a bevacizumab-based chemotherapeutic regimen	BMI <23.6	40	NA	34/46	NA	LV5FU + BEV: 6; FOLFIRI+BEV: 39; FOLFOX+BEV: 35	Liver: 23; Extrahepatic: 19; Both: 38	Wild: 40; Mutated: 12; Unknown: 28	24 (3–70)*	PFS	7
					BMI ≥23.6	40			NA				24 (3–70)*		
Hopirtean	2017	Romania	Cohort study	Patients with metastatic colorectal cancer, who followed bevacizumab based systemic therapy	BMI <27	77	55.5 (25–76)*	55/57	NA	NA	Liver: 63; Liver +lung: 15; Lung: 13; Other Sites: 21	NA	24 (3–70)*	PFS, OS	8
					BMI ≥27	35				NA			24 (3–70)*		
Kaidar-Person	2015	Israel	Cohort study	Only patients who were treated with bevacizumab as the first-line treatment for mCRC were included	BMI <25	68	NA	40/28	NA	NA	NA	NA	27.6 (1.1–107)*	PFS, OS	7
					25 ≤ BMI <30	70		42/28					27.6 (1.1–107)*		
					BMI >30	46		22/24					27.6 (1.1–107)*		
Patel	2015	Australia	Cohort study	MCRC patients who received bevacizumab-based chemotherapies	18.5 < BMI <25	63	60.9	36/27	NA	NA	NA	Wild: 46; Mutated: 17	>70	OS	8
					25 ≤ BMI <30	91	61.9	62/29	NA	NA	NA	Wild: 67; Mutated: 24	>70		
					30 ≤ BMI <35	35	59.9	24/11	NA	NA	NA	Wild: 22; Mutated: 13	>70		
					BMI ≥35	11	63.1	6/5	NA	NA	NA	Wild: 9; Mutated: 2	>70		

*Median (range); TTP, time to progression; FI + BEV, fluorouracil + irinotecan-based regimens; FO + BEV, fluoroura-cil + oxaliplatin-based regimens; F + BEV, fluorouracil-based regimens (5-FU or capecitabine); PFS, Progression-free survival; OS, overall survival; NA, Not applicable.

### Meta-analysis of the association between BMI and the prognosis of mCRC patients receiving bevacizumab

#### BMI < 30 kg/m^2^ VS BMI ≥ 30 kg/m^2^

##### Six-months OS

**Figure 2:**
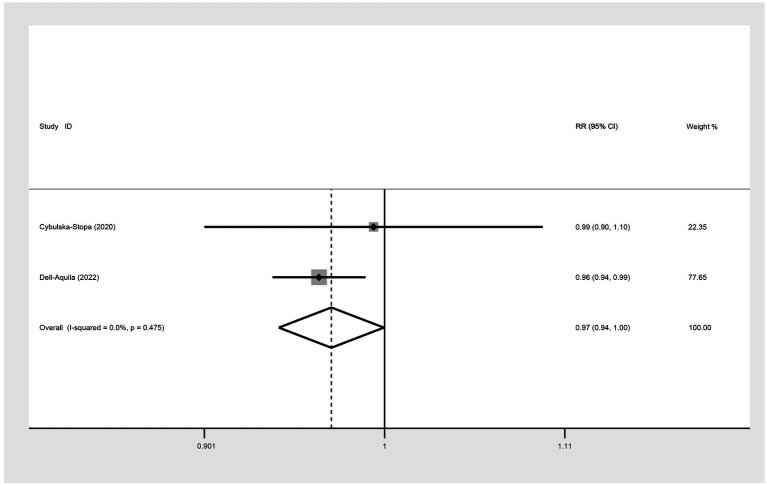
Meta-analysis of the association between BMI (BMI < 30 kg/m^2^ VS BMI ≥ 30 kg/m^2^) and 6-month OS of mCRC patients receiving bevacizumab.

Two included studies exploring the association between BMI and the prognosis of mCRC patients receiving bevacizumab treatment, comparing patients with a BMI of less than 30 kg/m^2^ (non-obese group) and those with a BMI of 30 kg/m^2^ or more (obese group) in terms of six-month OS. The result showed that I^2^ = 0.0%, indicating no observed heterogeneity among the studies, thereby a fixed-effect model was used for analysis. The result suggested that the 6-month OS for the non-obese group was slightly lower than that for the obese group (RR: 0.97, 95% CI: 0.94 to 1.00, *p* = 0.047) (Figure 2; Table 2).

**Table 2:** Meta-analysis of association between BMI and the prognosis of mCRC patients receiving bevacizumab plus chemotherapy.

BMI	Outcomes	WMD/RR/HR (95%CI)	*P*	I^2^
<30 kg/m^2^ VS >30 kg/m^2^	6-month OS			
	Overall	0.97 (0.94, 1.00)*	0.047	0.00%
	Sensitivity analysis	0.97 (0.94, 1.00)		
	1-year OS			
	Overall	0.92 (0.85, 1.00)*	0.054	0.00%
	Sensitivity analysis	0.92 (0.85, 1.00)		
	2-year OS			
	Overall	0.96 (0.62, 1.50)*	0.864	78.60%
	Sensitivity analysis	0.96 (0.62, 1.50)		
	5-year OS			
	Overall	0.80 (0.43, 1.52)*	0.498	0.00%
	Sensitivity analysis	0.80 (0.43, 1.52)		
	Median PFS			
	Overall	1.10 (−0.07, 2.28)**	0.065	0.00%
	Sensitivity analysis	1.10 (−0.07, 2.28)**		
	Median OS			
	Overall	3.93 (−2.91, 10.77)**	0.260	72.20%
	Sensitivity analysis	3.93 (−2.91, 10.77)**		
	PFS (HR)			
	Overall	0.91 (0.79, 1.05)***	0.195	0.00%
	Sensitivity analysis	0.91 (0.79, 1.05)		
>25 kg/m^2^ VS <25 kg/m^2^	PFS (HR)			
	Overall	0.99 (0.67, 1.46)***	0.423	69.40%
	Sensitivity analysis	0.99 (0.67, 1.46)		
	Obese vs. Normal	0.79 (0.41, 1.53)***	0.488	78.10%
	OS (HR)			
	Overall	1.00 (0.57, 1.74)***	0.987	77.40%
	Sensitivity analysis	1.00 (0.57, 1.74)		
	Obese vs. Normal	0.96 (0.34, 2.73)	0.939	86.50%
	Overweight vs. Normal	1.28 (0.44, 3.71)	0.645	80.50%
	Severely obese vs. Normal	1.00 (0.57, 1.74)	0.463	0.00%

BMI, body mass index; mCRC, metastatic colorectal cancer; OS, Overall survival; PFS, progression-free survival; *RR, risk ratio; **WMD, weighted mean difference; ***HR, hazard ratio; CI, confidence intervals.

##### One-year OS

**Figure 3:**
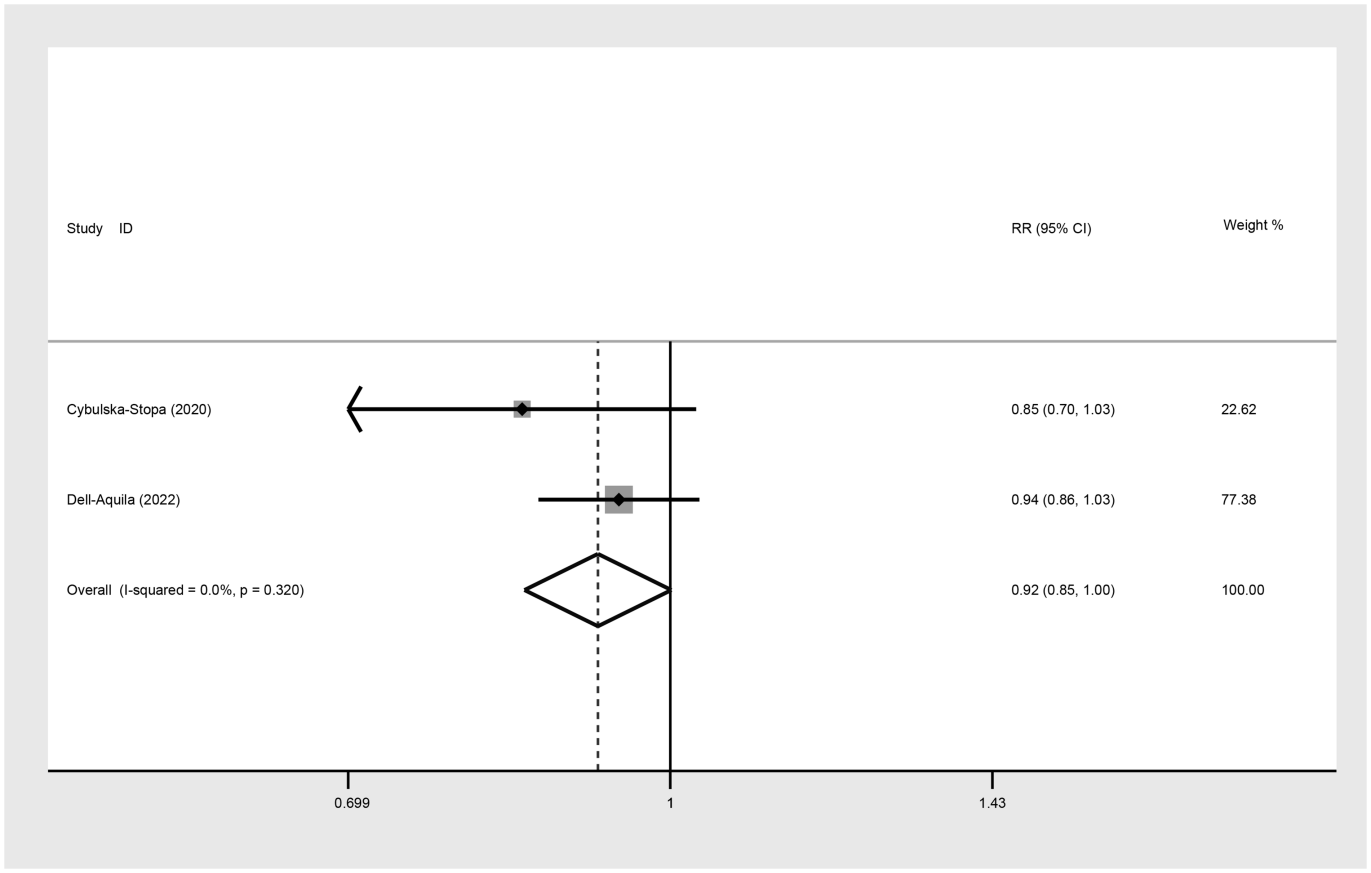
Meta-analysis of the association between BMI (BMI < 30 kg/m^2^ VS BMI ≥ 30 kg/m^2^) and 1-year OS of mCRC patients receiving bevacizumab.

Two studies were included to assess the 1-year OS between not obese and obese mCRC patients receiving bevacizumab. The heterogeneity was I^2^ = 0.0%, thus a fixed-effect model was used. Based on the findings, it cannot be conclusively stated that there was a difference in 1-year OS between the obese and none-obese groups (RR: 0.92, 95% CI: 0.85 to 1.00, *p* = 0.054) (Figure 3; Table 2).

##### Two-year OS

**Figure 4:**
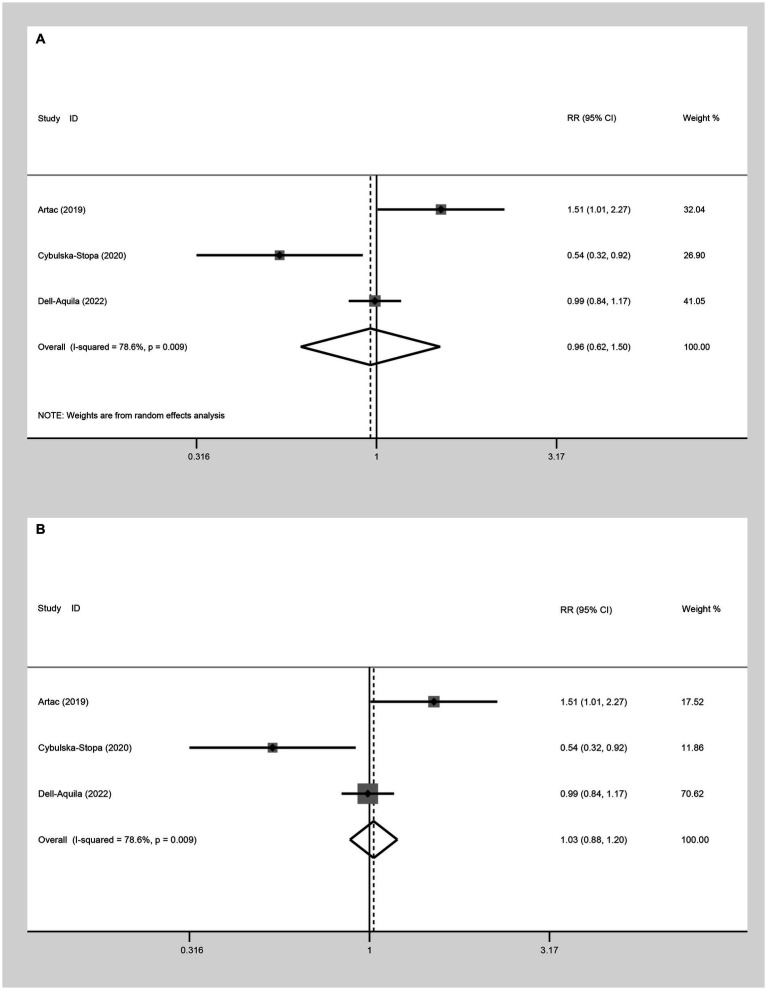
Meta-analysis of the association between BMI (BMI < 30 kg/m^2^ VS BMI ≥ 30 kg/m^2^) and 2-year OS of mCRC patients receiving bevacizumab. **(A)** The random-effect analysis. **(B)** The fixed-effect model analysis.

Three studies were incorporated to evaluate the 2-year OS comparing not-obese to the obese mCRC patients receiving bevacizumab treatment. The adoption of a random-effect model analysis (I^2^ = 78.6%) showed that there was no difference in 2-year OS between the obese and none-obese groups (RR: 0.96, 95% CI: 0.62 to 1.50, *p* = 0.864) (Figure 4A; Table 2). The fixed-effect model analysis also indicated no significant difference in the 2-year OS between obese and non-obese groups (RR: 1.03, 95% CI: 0.88 to 1.20, *p* = 0.728) (Figure 4B).

##### Five-year OS

**Figure 5:**
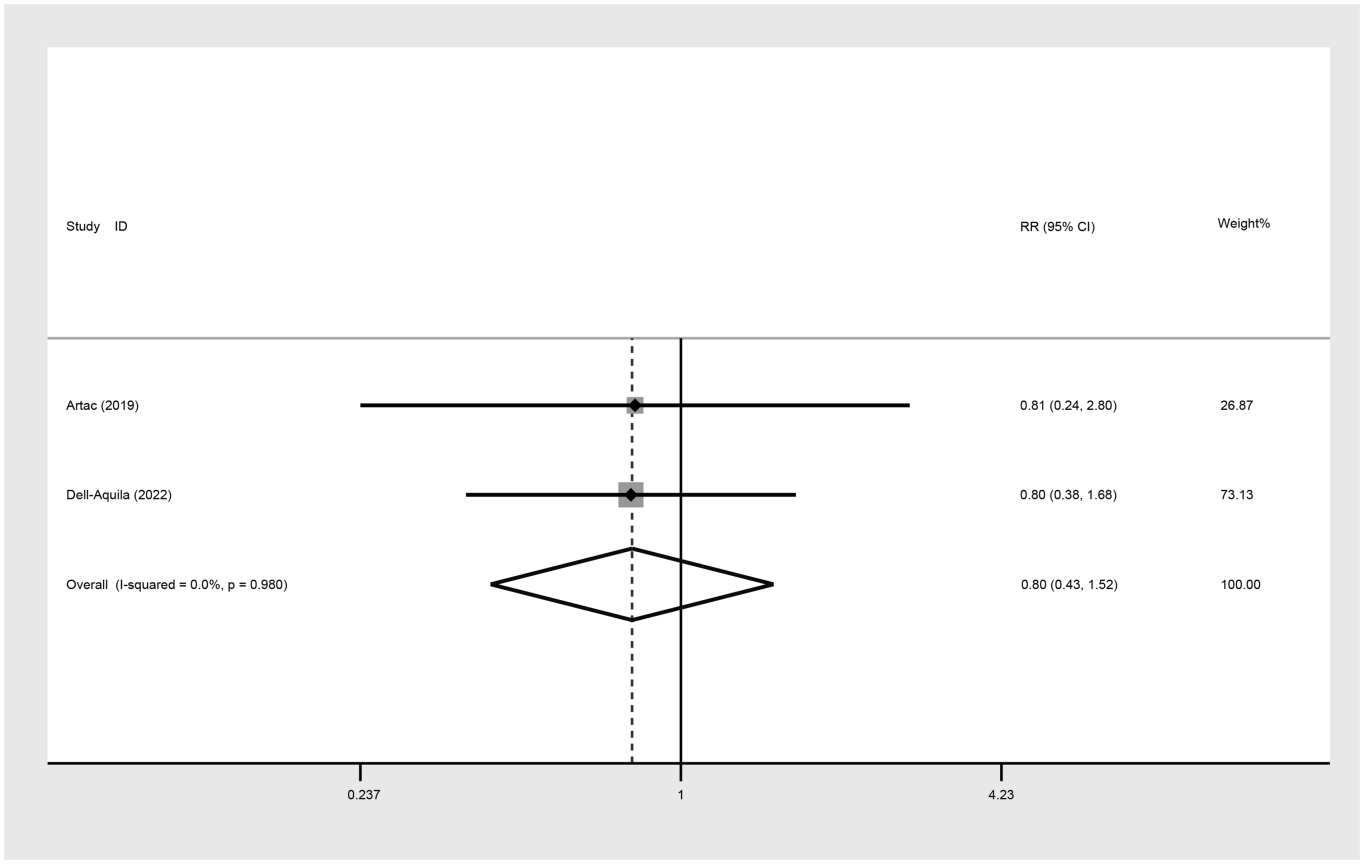
Meta-analysis of the association between BMI (BMI < 30 kg/m^2^ VS BMI ≥ 30 kg/m^2^) and 5-year OS of mCRC patients receiving bevacizumab.

In evaluating the 5-year OS comparison between non-obese and obese mCRC patients receiving bevacizumab treatment, two studies were included in the analysis. The heterogeneity index (I^2^) was determined to be 0.0%, leading to the application of a fixed-effect model. Based on the statistical outcomes, there did not exist a significant difference in 5-year OS outcomes between non-obese and obese populations (RR: 0.80, 95% CI: 0.43 to 1.52, *p* = 0.498) (Figure 5; Table 2).

##### Median PFS

**Figure 6:**
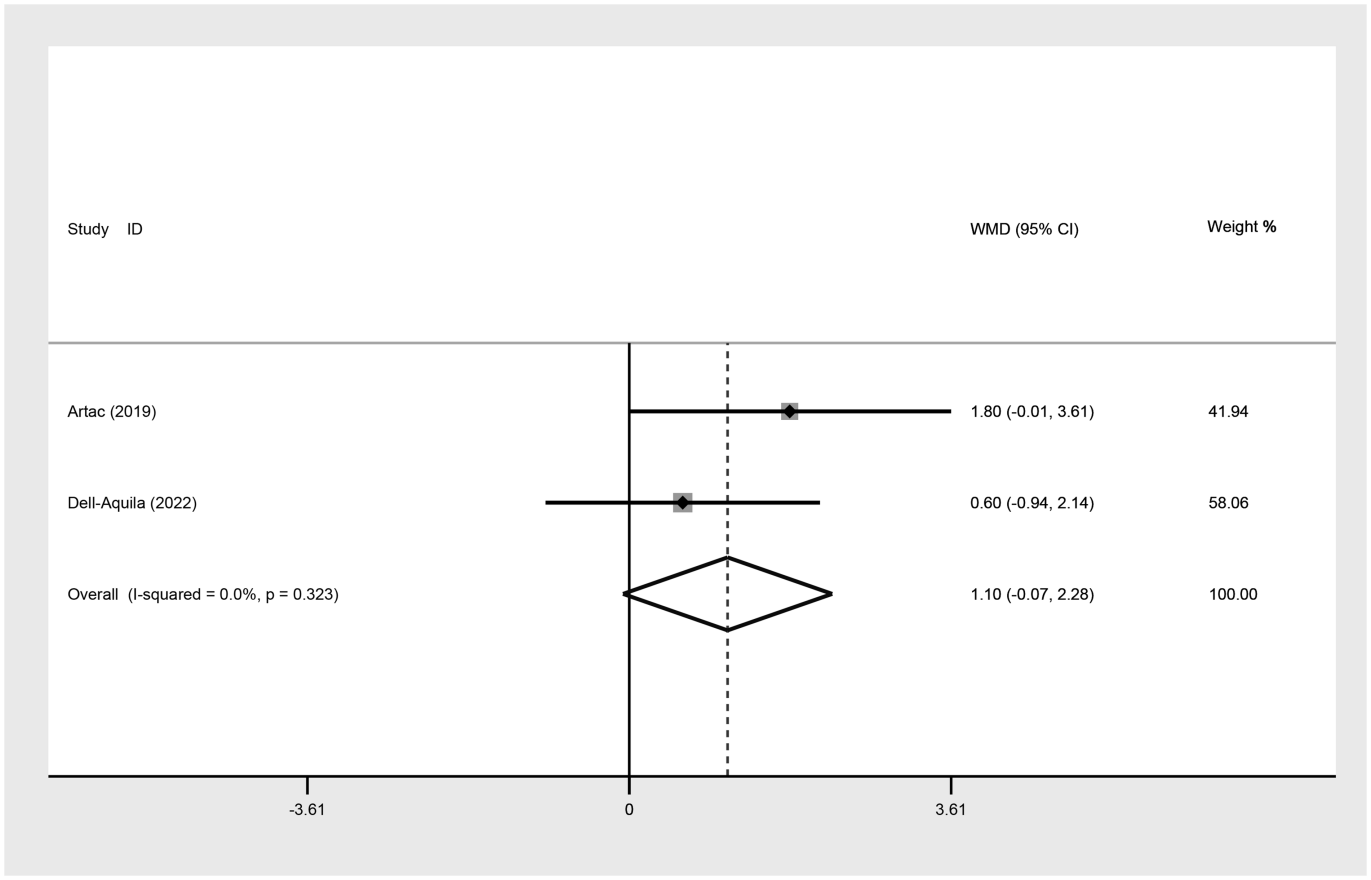
Meta-analysis of association between BMI (BMI < 30 kg/m^2^ VS BMI ≥ 30 kg/m^2^) and median PFS of mCRC patients receiving bevacizumab.

For the comparison of median PFS between not obese and obese mCRC patients receiving bevacizumab treatment, two studies were incorporated into the analysis. The results suggested that there was no significant difference in the median PFS between the obese and not obese groups (WMD: 1.10, 95% CI: −0.07 to 2.28, *p* = 0.065) (Figure 6; Table 2).

##### Median OS

**Figure 7:**
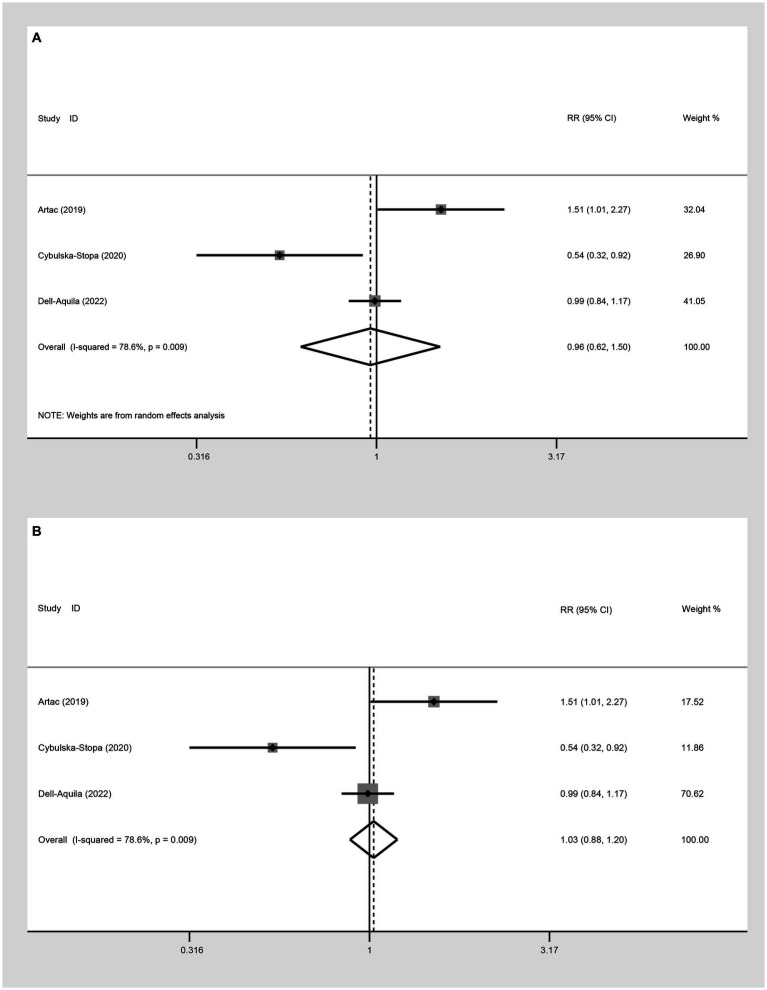
Meta-analysis of association between BMI (BMI < 30 kg/m^2^ VS BMI ≥ 30 kg/m^2^) and median OS of mCRC patients receiving bevacizumab. **(A)** The random-effect analysis. **(B)** The fixed-effect model analysis.

In the assessment of median OS between the not obese and obese mCRC patients receiving bevacizumab treatment, two studies were analyzed. The heterogeneity among these studies indicated by an I^2^ value of 72.2%. The adoption of a random-effects model analysis (WMD: 3.93, 95% CI: −2.91 to 10.77, *p* = 0.260) (Figure 7A; Table 2) and fixed-effect model analysis (WMD: 3.21, 95% CI: −0.26 to 6.68, *p* = 0.069) (Figure 7B) both showed that there was no difference in the median OS between the obese and not obese groups.

##### PFS (HR)

**Figure 8:**
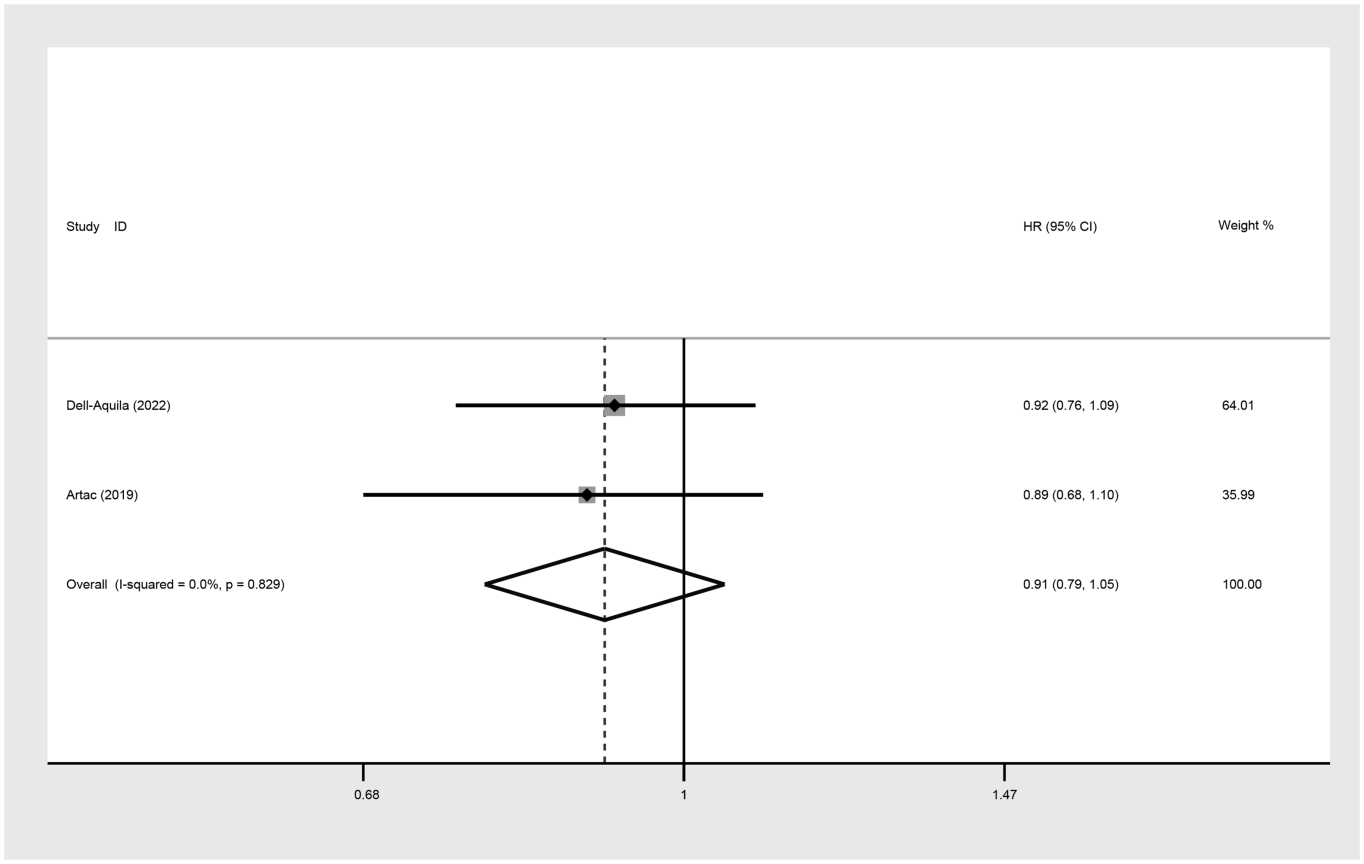
Meta-analysis of the association between BMI (BMI < 30 kg/m^2^ VS BMI ≥ 30 kg/m^2^) and PFS (HR) of mCRC patients receiving bevacizumab.

The comparison of PFS between not obese and obese mCRC patients receiving bevacizumab treatment involved the analysis of two studies. The heterogeneity among these studies was quantified at I^2^ = 0.0%, which led to the utilization of a fixed-effect model. The result suggested that the PFS was lower in the non-obese group compared to the obese group (HR: 0.91, 95% CI: 0.79 to 1.05, *p =* 0.195) (Figure 8; Table 2).

#### BMI ≥ 25 kg/m^2^ VS BMI < 25 kg/m^2^

##### PFS (HR)

**Figure 9:**
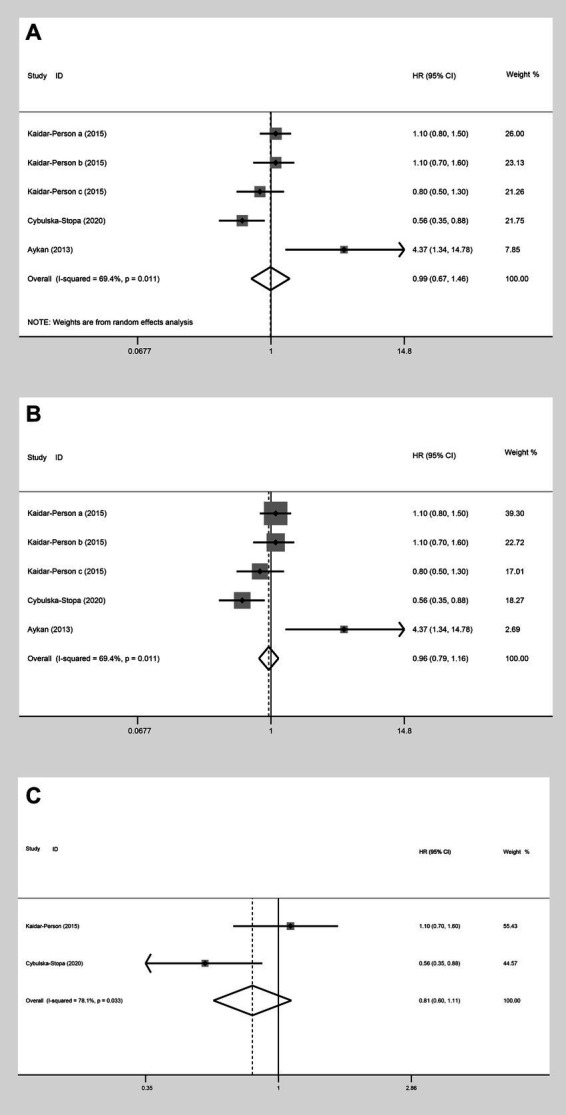
Meta-analysis of the association between BMI (BMI ≥ 25 kg/m^2^ VS BMI < 25 kg/m^2^) and PFS (HR) of mCRC patients receiving bevacizumab. **(A)** The random-effect analysis. **(B)** The fixed-effect model analysis. **(C)** Subgroup result of the fixed-effect model analysis.

In the analysis of PFS (HR) comparing individuals with a BMI ≥ 25 kg/m^2^ to those with a BMI < 25 kg/m^2^, three studies were included. The random-effect model analysis (HR: 0.99, 95% CI: 0.67 to 1.46, *p* = 0.423, I^2^ = 69.4%) (Figure 9A; Table 2) and the fixed-effect model analysis (HR: 0.96, 95% CI: 0.79 to 1.16, *p* = 0.654) (Figure 9B) both indicated no significant difference in PFS (HR) between the groups.

Upon further analyses of the group with BMI ≥ 25 kg/m^2^ and BMI < 25 kg/m^2^ into the subgroup of obese versus normal, both random-effect model analysis (HR: 0.79, 95% CI: 0.41 to 1.53, *p* = 0.488, I^2^ = 78.10%) (Table 2) and fixed-effect model analysis (HR: 0.81, 95% CI: 0.60 to 1.11, *p* = 0.190) (Figure 9C) showed no statistically significant differences in PFS between the groups.

##### OS (HR)

**Figure 10:**
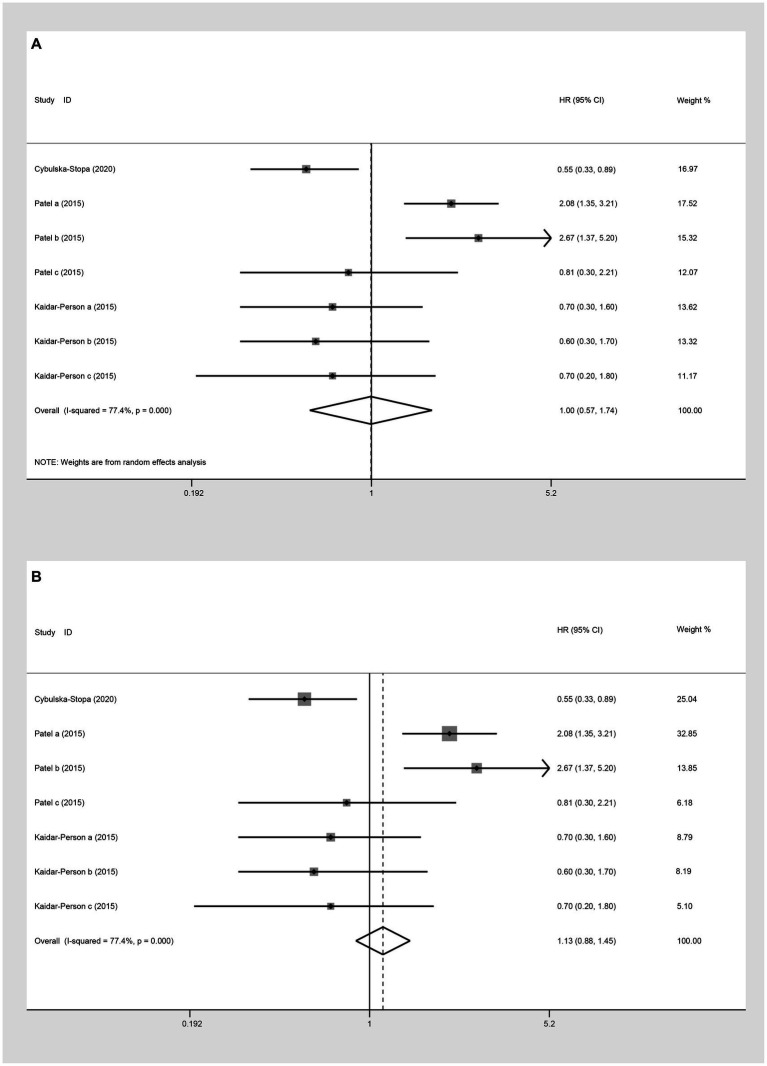
Meta-analysis of the association between BMI (BMI ≥ 25 kg/m^2^ VS BMI < 25 kg/m^2^) and OS (HR) of mCRC patients receiving bevacizumab. **(A)** The random-effect analysis. **(B)** The fixed-effect model analysis.

Three studies assessed OS (HR) for groups classified as BMI ≥ 25 kg/m^2^ versus BMI < 25 kg/m^2^. The heterogeneity test showed that I^2^ = 77.4%. The results of the random-effect model analysis (HR: 1.00, 95% CI: 0.57 to 1.74, *p* = 0.987) (Figure 10A; Table 2) and (HR: 1.13, 95% CI: 0.88 to 1.45, *p* = 0.333) indicated no difference in OS (HR) between the groups (Figure 10B). Upon further analysis using the random-effect model analysis, dividing the BMI > 25 kg/m^2^ versus BMI < 25 kg/m^2^ group into subgroups: obese versus normal, overweight versus normal, and severely obese versus normal. These findings suggested that there were no significant differences in OS (HR) among obese versus normal (HR: 0.96, 95% CI: 0.34 to 2.73, *p* = 0.939), overweight versus normal (HR: 1.28, 95% CI: 0.44 to 3.71, *p* = 0.645), and severely obese versus normal (HR: 1.00, 95% CI: 0.57 to 1.74, *p* = 0.463) groups (Table 2).

### A systematic review of the association between BMI and the prognosis of mCRC patients receiving bevacizumab

#### 6-month OS

A study conducted by Cybulska-Stopa et al. (17) in 2020 demonstrated that there was no statistically significant difference in the 6-month OS between the normal BMI group and the overweight (RR: 0.98, 95% CI: 0.89 to 1.08, *p* = 0.720), obese (RR: 0.98, 95% CI: 0.88 to 1.10, *p* = 0.772), and non-normal (RR: 0.98, 95% CI: 0.89 to 1.07, *p* = 0.601) groups.

#### One-year OS

In a study by Cybulska-Stopa et al. (17), the analysis showed no statistically significant difference in the 1-year OS between the normal BMI group and the overweight (RR: 0.91, 95% CI: 0.72 to 1.15, *p* = 0.443), obese (RR: 0.80, 95% CI: 0.63 to 1.03, *p* = 0.08), and non-normal (RR: 0.87, 95% CI: 0.70 to 1.08, *p* = 0.220) groups.

#### Five-year OS

In a 2017 study by Hopirtean et al. (24), no statistically significant difference was observed in the 5-year OS between patients with a BMI less than 27 and those with a BMI greater than 27 kg/m^2^ (RR: 1.12, 95% CI: 0.63 to 1.99, *p* = 0.710).

#### Median PFS

The study by Aykan et al. (21) highlighted that the median PFS was significantly higher in the normal BMI group compared to the non-normal group (WMD: 5.70, 95% CI: 3.71 to 7.70, *p* = 0.001). The research by Guiu et al. (23) revealed that patients with lower BMI (less than 23.6) values had a longer median PFS. Similarly, the study by Hopirtean et al. (24) indicated that patients with a BMI of less than 27 had a significantly longer median PFS compared to those with a BMI greater than 27. In a 2018 study by Cong et al. (22), no significant difference was found in median PFS between groups with high △ BMI and low △BMI.

#### Median OS

In the study by Cong et al. (22), patients with a high △BMI had a higher median OS compared to those with a low △BMI.

## Discussion

This systematic review and meta-analysis included 9 eligible cohort studies to evaluate the impact of BMI on the prognosis of patients with mCRC receiving bevacizumab. The findings of our study suggested that obese mCRC patients had a slightly better OS at 6 months compared to the non-obese group. In addition, between the groups of obese and non-obese mCRC patients, there were no observable differences in terms of one-year, two-year, and five-year OS, as well as in the PFS and median OS.

Our analysis revealed that, after six months, patients with mCRC receiving bevacizumab who were obese demonstrated an improvement in OS compared to those who were not obese. Previous studies have confirmed the positive impact of obesity on the outcomes of mCRC patients with or without bevacizumab treatment. A pooled analysis of individual data from 2,085 patients enrolled in eight FFCD first-line mCRC trials revealed that obesity and overweight compared with normal weight had a positive effect on OS in patients with mCRC (13). The ARCAD analysis of 21,149 patients enrolled in 25 first-line clinical trials in mCRC showed that low BMI was associated with an increased risk of progression and death (27). In an analysis, patients with mCRC treated with chemotherapy and bevacizumab in second-line treatment with higher BMI compared with normal weight patients had better prognosis in terms of OS (17). The positive impact of obesity on survival patients with mCRC receiving bevacizumab could be influenced by several potential clinical mechanisms. Bevacizumab is a monoclonal antibody that targets VEGF, a key mediator of angiogenesis (28). Obesity is associated with increased levels of VEGF (15), which could potentially lead to a different response to VEGF inhibition in obese versus non-obese patients. Leptin, a hormone predominantly produced by adipose tissue, is elevated in obese individuals (29). Since leptin is a positive modulator of both the innate and the adaptive immune system, it may contribute to the increased immune response stimulated by immunotherapy in cancer patients and may be proposed as a good actor in cancer (30). All in all, our result suggests that obesity might not universally be a negative prognostic factor in the context of specific treatments and could prompt a reevaluation of treatment protocols for obese patients, highlighting the need for personalized medicine approaches, considering patient-specific factors, including BMI, in the decision-making process for cancer treatment.

A study assessing the BMI at diagnosis and survival duration of colon cancer patients enrolled in adjuvant chemotherapy clinical trials found that being underweight was independently associated with a poor prognosis in patients treated with adjuvant chemotherapy trials (31). In a pooled analysis of FFCD trials, OS and PFS were significantly shorter in OS and PFS were significantly shorter in mCRC patients with BMI <18.5 kg/m^2^ (13). A meta-analysis of prospective cohort studies found that being underweight after diagnosis is significantly associated with an increased risk of all-cause mortality in colorectal cancer (32). The findings underscore the critical association between underweight status and adverse outcomes in colorectal cancer patients. However, the underweight population could not be included in the study due to literature limitations. This limitation suggests that further research is imperative to gain a comprehensive understanding of the impact of underweight status on mCRC prognosis.

Our meta-analysis on the impact of BMI on the prognosis of mCRC patients receiving bevacizumab sheds light on the complex interplay between BMI and cancer treatment outcomes. First, by exploring the nuanced relationship between BMI and the effectiveness of targeted therapies, this study contributes to a growing body of evidence suggesting that patient-specific factors, such as BMI, can significantly influence treatment efficacy and survival rates. This underscores the importance of considering individual patient characteristics in treatment planning. Second, recognizing the impact of BMI on treatment outcomes highlights the importance of nutrition and weight management in cancer treatment in nursing care. Understanding this relationship can guide nurses in developing personalized care plans that consider nutritional status and body weight as key factors in patient management. It emphasizes the importance of a holistic approach to patient care, including nutritional counseling and weight management strategies, to potentially enhance treatment efficacy and improve patient outcomes. Furthermore, studies to explore the relationship between BMI and prognosis in patients with mCRC treated with bevacizumab may provide an evidence base for the development of cancer treatment guidelines. Our study may help develop more precise treatment strategies and optimize patient outcomes.

The limitations of this study should be mentioned. First, the limitation of including a small number of studies in the analysis could lead to less robust conclusions due to potential sample bias and reduced statistical power. This limitation makes it challenging to generalize the findings to the broader mCRC patient population receiving bevacizumab. Second, some WMD/RR/HR values in a meta-analysis were close to 1. A WMD/RR/HR nears 1 may indicate that the study lacks sufficient power to detect a true effect. In addition, when WMD/RR/HR values are close to 1 across different studies, it may mask underlying heterogeneity. This could mean that there were important differences between studies that were not being captured. Third, the high heterogeneity observed in some results could be attributed to variations in chemotherapy regimens combined with bevacizumab and different KRAS mutation types among the studies. This variation suggests that the effects of bevacizumab might not be uniform across all treatment scenarios and genetic profiles, indicating a complex interaction between the drug’s efficacy and specific patient or tumor characteristics. However, due to the limitations of the studies included, it was not possible to fully identify the sources of this heterogeneity, highlighting a gap in the current understanding that warrants further investigation. Fouth, including only English-language studies could introduce a language bias, potentially excluding relevant research published in other languages. This limitation might affect the comprehensiveness and applicability of the findings to diverse populations. Fifth, the exclusion of underweight patient groups due to literature limitations might have restricted the study’s ability to fully explore the impact of BMI across the entire spectrum of body weight, potentially overlooking how bevacizumab’s efficacy varies in this specific population. More studies are needed to provide a more comprehensive understanding of bevacizumab’s efficacy in mCRC treatment across various patient populations.

## Conclusion

In our analysis, non-obese patients with mCRC treated with bevacizumab had a lower OS rate at 6 months compared to the obese group. These insights are clinically significant as they highlight the importance of considering body weight in the management of mCRC and may guide future research into personalized treatment strategies. More research is needed to confirm our findings.

## Data availability statement

The raw data supporting the conclusions of this article will be made available by the authors, without undue reservation.

## Author contributions

XL: Conceptualization, Data curation, Formal analysis, Investigation, Methodology, Supervision, Writing – original draft, Writing – review & editing. CT: Conceptualization, Data curation, Formal analysis, Investigation, Project administration, Supervision, Writing – original draft. YY: Data curation, Formal analysis, Investigation, Methodology, Writing – review & editing. HL: Conceptualization, Supervision, Writing – original draft, Writing – review & editing.
