# Supplementary figures and images for: Effect of body mass index on survival in patients with metastatic colorectal cancer receiving chemotherapy plus bevacizumab: a systematic review and meta-analysis
Source: Front Nutr. 2024 Jul 16;11:1399569. doi: 10.3389/fnut.2024.1399569 (PMC11288195; doi:10.3389/fnut.2024.1399569)

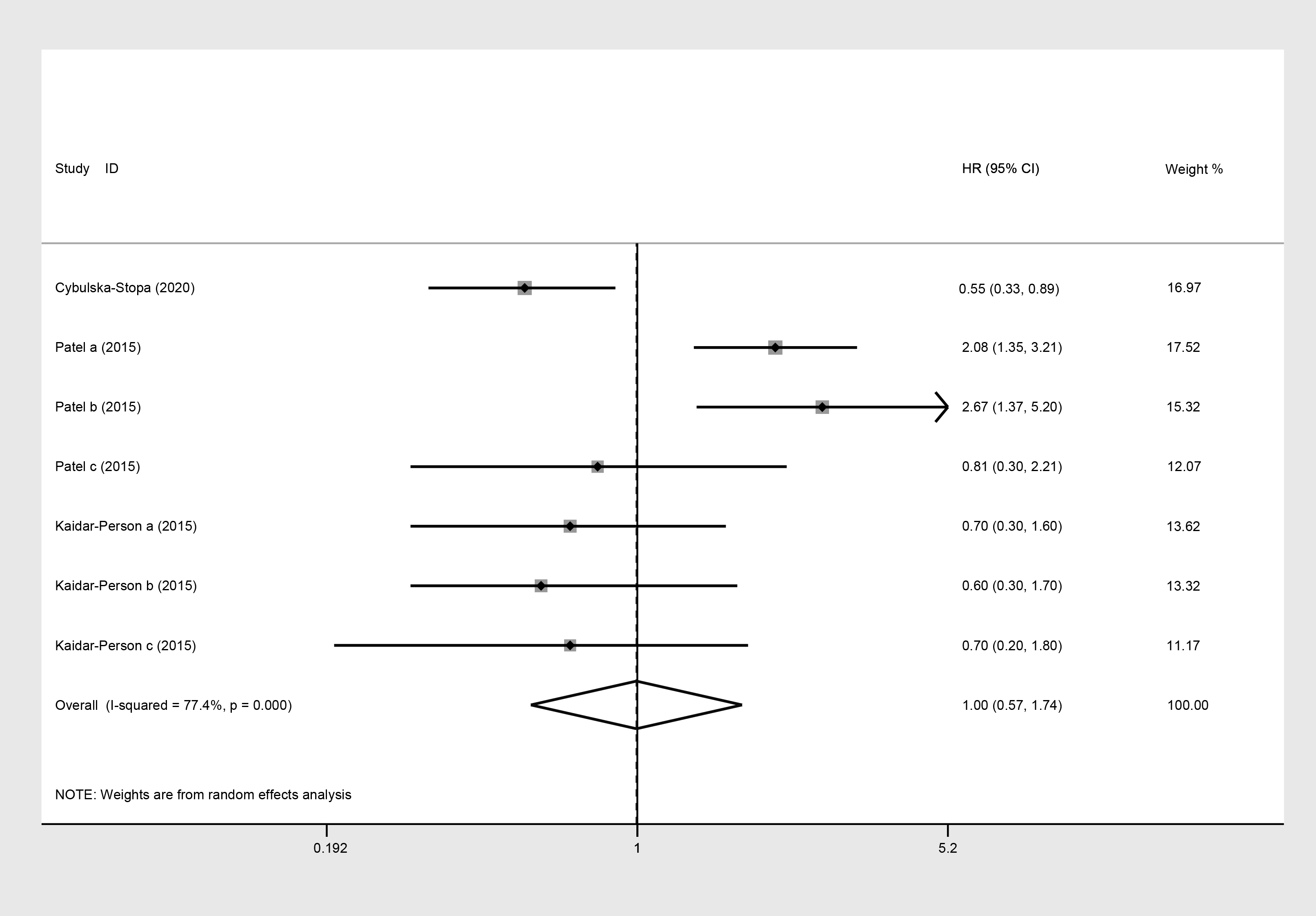

Supplement: Supplementary file 1 [file Image_1.TIF]

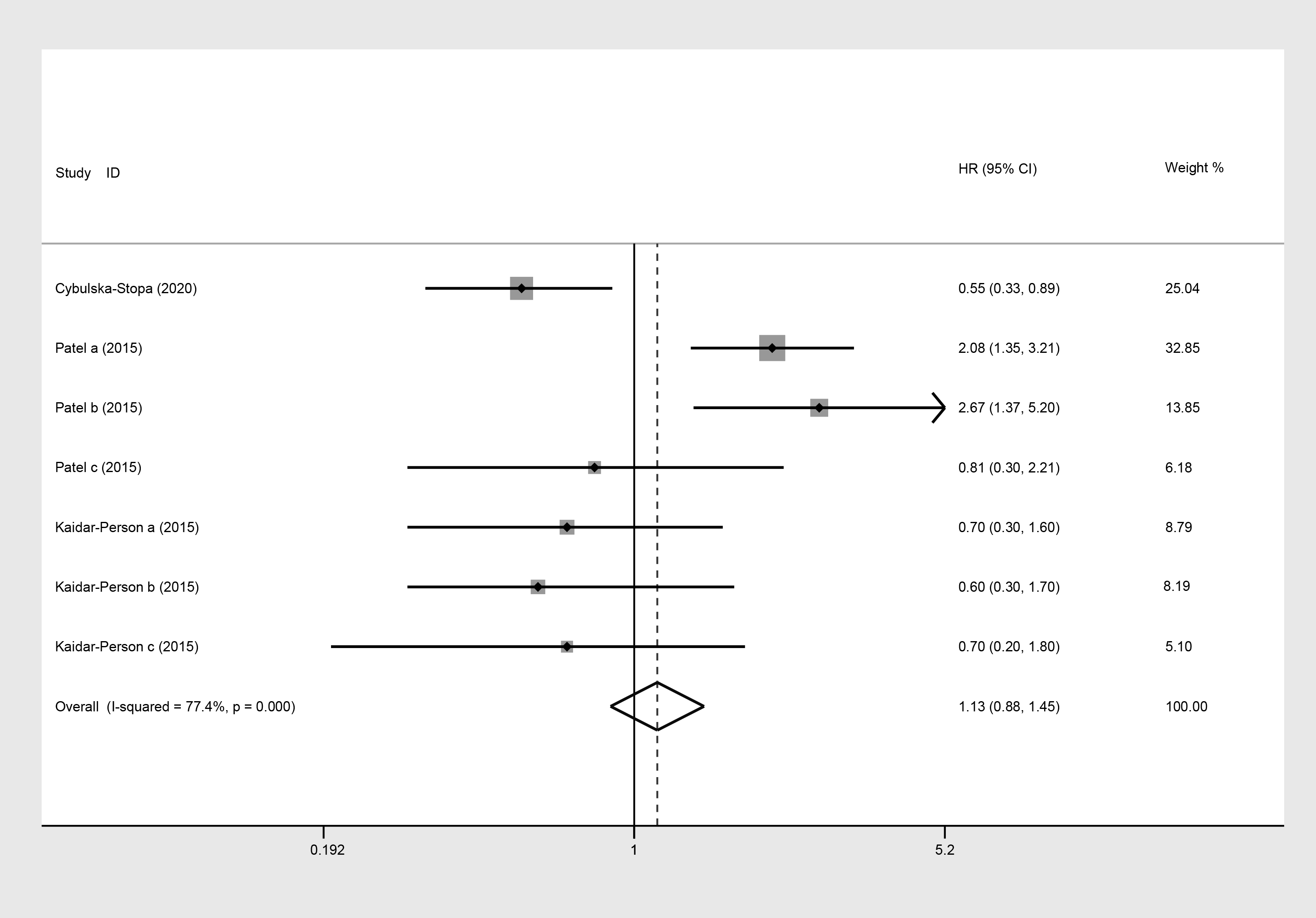

Supplement: Supplementary file 2 [file Image_2.TIF]
