# Supplementary material for: Effect of body mass index on survival in patients with metastatic colorectal cancer receiving chemotherapy plus bevacizumab: a systematic review and meta-analysis
Source: Front Nutr. 2024 Jul 16;11:1399569. doi: 10.3389/fnut.2024.1399569 (PMC11288195; doi:10.3389/fnut.2024.1399569)
Supplement: Supplementary file 3 [file Table_1.DOC]

The Embase search strategy: ('colorectal neoplasms':ab,ti OR 'colorectal neoplasm':ab,ti OR 'neoplasm, colorectal':ab,ti OR 'neoplasms, colorectal':ab,ti OR 'colorectal tumors':ab,ti OR 'colorectal tumor':ab,ti OR 'tumor, colorectal':ab,ti OR 'tumors, colorectal':ab,ti OR 'colorectal cancer':ab,ti OR 'cancer, colorectal':ab,ti OR 'cancers, colorectal':ab,ti OR 'colorectal cancers':ab,ti OR 'colorectal carcinoma':ab,ti OR 'carcinoma, colorectal':ab,ti OR 'carcinomas, colorectal':ab,ti OR 'colorectal carcinomas':ab,ti) and ('body mass index':ab,ti OR 'index, body mass':ab,ti OR 'quetelet index':ab,ti OR 'index, quetelet':ab,ti OR 'quetelets index':ab,ti OR bmi:ab,ti)

The Web of Science search strategy: (((((((TS=(Body Mass Index)) OR TS=(Index, Body Mass)) OR TS=(Quetelet Index)) OR TS=(Index, Quetelet)) OR TS=(Quetelet's Index)) OR TS=(Quetelets Index)) OR TS=(BMI)) and ((((((((TS=(Colorectal Neoplasm*)) OR TS=(Neoplasm*, Colorectal)) OR TS=(Colorectal Tumor*)) OR TS=(Tumor*, Colorectal)) OR TS=(Colorectal Cancer*)) OR TS=(Cancer*, Colorectal)) OR TS=(Colorectal Carcinoma*)) OR TS=(Carcinoma*, Colorectal))

The Cochrane search strategy:

((Colorectal Neoplasm* or Neoplasm*, Colorectal or Colorectal Tumor* or Tumor*, Colorectal or Colorectal Cancer* Cancer*, Colorectal or Colorectal Carcinoma* or Carcinoma*, Colorectal):ti,ab,kw) and ((Body Mass Index or Index, Body Mass or Quetelet Index or Index, Quetelet or Quetelet's Index or Quetelets Index or BMI):ti,ab,kw)
